# Supplementary material for: Development of a computer-aided design software for the quantitative evaluation of aesthetic damage
Source: PLoS One. 2019 Dec 18;14(12):e0226322. doi: 10.1371/journal.pone.0226322 (PMC6919621; doi:10.1371/journal.pone.0226322)
Supplement: S1 Code site — 1. (ZIP) [file pone.0226322.s001.zip › [EESC_jr][Projeto]Relatorio_07_21/vendors/select2/docs/_includes/examples/matcher.html]

# Customizing how results are matched

Unlike other dropdowns on this page, this one matches options only if
the term appears in the beginning of the string as opposed to anywhere:

This custom matcher uses a
compatibility module that is
only bundled in the
full version of Select2. You also
have the option of using a
more complex matcher.

{% highlight js linenos %}
function matchStart (term, text) {
if (text.toUpperCase().indexOf(term.toUpperCase()) == 0) {
return true;
}
return false;
}
$.fn.select2.amd.require(['select2/compat/matcher'], function (oldMatcher) {
$(".js-example-matcher-start").select2({
matcher: oldMatcher(matchStart)
})
});
{% endhighlight %}
